# Editorial: Using omics to study leprosy, tuberculosis, and other mycobacterial diseases

**DOI:** 10.3389/fcimb.2022.1043635

**Published:** 2022-09-29

**Authors:** Cristiana Santos de Macedo, Luis Caetano Martha Antunes, Keshar Kunja Mohanty

**Affiliations:** ^1^ Center for Technological Development in Health, Oswaldo Cruz Foundation, Rio de Janeiro, Brazil; ^2^ Oswaldo Cruz Institute, Oswaldo Cruz Foundation, Rio de Janeiro, Brazil; ^3^ Department of Immunology, Indian Council of Medical Research (ICMR)-National JALMA Institute for Leprosy and Other Mycobacterial Diseases, Agra, India

**Keywords:** *Mycobacterium leprae*, *Mycobacterium tuberculosis*, non-tuberculous mycobacteria (NTM), host-pathogen interaction, molecular signatures

Diseases caused by *Mycobacterium* species have always been prominent public health problems. Notwithstanding, many aspects of basic bacterial biology, pathogenesis, transmission, and immune regulation remain unknown. Of the various important mycobacterial human diseases, tuberculosis represents the largest global threat, as it is currently the second most deadly infectious disease, behind COVID-19 only; In 2020, an estimated 1.5 million people died from tuberculosis, a preventable and treatable disease (World Health Organization, 2021a). As the second player between mycobacterial diseases, leprosy affects neglected populations in low- and middle-income countries, causing permanent disabilities if left untreated. The COVID-19 pandemic reduced the detection and reporting of new leprosy cases by 37% (World Health Organization, 2021b), which will have a negative impact on disease control for years to come. Besides *M. tuberculosis* and *M. leprae*, the causative agents of tuberculosis and leprosy, respectively, non-tuberculous mycobacteria (NTM) are gaining importance as pathogens, as increasing numbers of infections are being reported worldwide (Ratnatunga et al., 2020).

In all cases of mycobacterial diseases cited above, available diagnostic methods are mostly outdated, therapeutic options are limited, and antibiotic resistance poses a significant challenge to infection treatment and disease control. Therefore, new methods to study these microbes and the diseases they cause are urgently needed. Besides generating new information about the basic functioning of the organisms and how they interact with their hosts, new tools and methods of study have the potential to unfold into new diagnostic methods and therapeutic targets. To that end, the so called ‘omics’ methods have been increasingly used for both basic and translational scientific purposes in the field of infectious diseases. Omics is a growing field of research that encompasses many areas, like genomics, transcriptomics, proteomics, metabolomics, and lipidomics. As mentioned above, omics studies may bring invaluable information about molecular signatures in bacteria and host-pathogen relationships during mycobacterial diseases, and can also provide a foundation for the development of new diagnostic and therapeutic tools.

Given the importance of mycobacterial diseases and the potential of the omics fields in addressing important, unanswered questions in infectious diseases, this Research Topic compiled four original articles and one review to provide the reader with an outlook on the role of omics in mycobacterial research. Ahamad et al. reviewed the wealth of literature covering the whole array of multi-omics strategies to study mycobacterial diseases. The authors have put forth models to discover potential host biomarkers for the diagnosis of these diseases using various current omics technologies, and discussed the techniques used in genomics, transcriptomics, proteomics, metabolomics, and lipidomics, as well as current progresses in different fields of research, such as bacterial pathogenicity, host immune and inflammatory responses, biomarkers, diagnostics, and antibiotic resistance, among others.

Ojo et al. addressed the changes in the transcriptome of *M. leprae* during the transition of *in vivo* growth to *ex vivo* stationary phase, which reflected the metabolic adaptations to the environment, such as the downregulation of genes involved in beta-oxidation due to the lack of lipids in the *ex vivo* medium. *M. leprae* is, to date, not culturable *in vitro* or in relevant animal models of the disease, and therefore most of the information about host-pathogen interactions is derived from the natural host, humans. The study by Ojo et al. brought important insights on *M. leprae* metabolic requirements, which are needed for the pursuit of axenic growth of this microorganism, currently the most important barrier for leprosy research.

Ferreira et al. assessed gene expression patterns in the skin of lepromatous leprosy patients before and after multidrug therapy (MDT) treatment and observed a down regulation on genes related to chemokine and IFN pathways after MDT. Gene expression between patients who responded to the treatment (bacterial index reduction higher than 1 log) and those who were considered non-responders (bacterial index reduction lower than 1 log) was also compared, showing that genes upregulated in MDT responders were mainly involved in skin homeostasis and development as well as fat-soluble vitamin metabolism. As such, authors have found a molecular signature associated with a better response to MDT. This study sheds some light on the fate of immune responses and various mediators playing a role in host-pathogen dynamics. These differences point to pathways that could be explored for the development of new drugs to improve the efficacy of MDT.

Good epidemiological vigilance requires the constant improvement of molecular epidemiology tools to study the dynamics of disease transmission. Tuberculosis is a major public health concern in Mexico. Chiapas, a Southern state located in the Mexico-Guatemala border has high tuberculosis mortality and morbidity rates. Molina-Torres et al. reported the characterization of isolates from this region using spoligotyping and whole genome sequencing. This study is the first to report the genetic diversity of *M. tuberculosis* within the context of a Mexican-Amerindian population.

Although the host context of infectious diseases has been extensively studied, it was relatively recently that the microbiota colonizing the host has started to be taken into consideration in infectious disease research. Ding et al. used metagenomics to characterize the lung microbiota of tuberculosis patients. By comparing the microbial composition of bronchoalveolar lavage fluid of patients with bacteriologically-confirmed tuberculosis versus those who were clinically diagnosed with tuberculosis but where the pathogen could not be detected, authors report microbial groups that are significantly associated with each group. Their results add to the growing body of evidence that the microbiota can be used as a marker of health/disease and may have important yet undefined roles in infection dynamics.

The aforementioned articles are only a small demonstration of the potential of omics to unveil novel aspects of mycobacterial basic biology and host interactions. We invite the reader to delve into this promising and fascinating field of research using the articles in this Research Topic, with hopes that this will spark an even greater interest in the field.

## Author contributions

CSM, LA, and KKM contributed to the writing of the manuscript and approved the submitted version.

## Funding

CSM is funded by grants from FAPERJ (E-26/010.001453/2019, E-26/010.002231/2019, E-26/211.401/2021, E-26/211.587/2021), PRONEX/CNPq/FAPERJ (E-26/010.000172/2020), and Inova Fiocruz (VPPCB-007-FIO-18). LCMA is funded by grants from CNPq (303843/2017-9), FAPERJ (E-26/202.705/2018, E-26/210.209/2018, E-26/010.002155/2019, SEI-260003/001213/2020, E-26/211.554/2019, E-26/010.001280/2016) and CAPES (Finance code 001). KKM thanks the Indian Council of Medical Research, New Delhi, India. KKM is working in ICMR- National JALMA Institute for Leprosy and Other Mycobacterial Diseases, Agra, under the organization, Indian Council of Medical Research, Department of Health Research, Ministry of Health and Family Welfare, Government of India, New Delhi, India.

## Conflict of interest

The authors declare that the research was conducted in the absence of any commercial or financial relationships that could be construed as a potential conflict of interest.

## Publisher’s note

All claims expressed in this article are solely those of the authors and do not necessarily represent those of their affiliated organizations, or those of the publisher, the editors and the reviewers. Any product that may be evaluated in this article, or claim that may be made by its manufacturer, is not guaranteed or endorsed by the publisher.
